# LM-DTI: a tool of predicting drug-target interactions using the node2vec and network path score methods

**DOI:** 10.3389/fgene.2023.1181592

**Published:** 2023-05-09

**Authors:** Jianwei Li, Yinfei Wang, Zhiguang Li, Hongxin Lin, Baoqin Wu

**Affiliations:** ^1^ School of Artificial Intelligence, Institute of Computational Medicine, Hebei University of Technology, Tianjin, China; ^2^ School of Electronic and Information Engineering, Hebei University of Technology, Tianjin, China

**Keywords:** drug-target interaction, heterogeneous information network, node2vec, network path score, XGBoost

## Abstract

**Introduction:** Drug-target interaction (DTI) prediction is a key step in drug function discovery and repositioning. The emergence of large-scale heterogeneous biological networks provides an opportunity to identify drug-related target genes, which led to the development of several computational methods for DTI prediction.

**Methods:** Considering the limitations of conventional computational methods, a novel tool named LM-DTI based on integrated information related to lncRNAs and miRNAs was proposed, which adopted the graph embedding (node2vec) and the network path score methods. First, LM-DTI innovatively constructed a heterogeneous information network containing eight networks composed of four types of nodes (drug, target, lncRNA, and miRNA). Next, the node2vec method was used to obtain feature vectors of drug as well as target nodes, and the path score vector of each drug-target pair was calculated using the DASPfind method. Finally, the feature vectors and path score vectors were merged and input into the XGBoost classifier to predict potential drug-target interactions.

**Results and Discussion:** The 10-fold cross validations evaluate the classification accuracies of the LM-DTI. The prediction performance of LM-DTI in AUPR reached 0.96, which showed a significant improvement compared with those of conventional tools. The validity of LM-DTI has also been verified by manually searching literature and various databases. LM-DTI is scalable and computing efficient; thus representing a powerful drug relocation tool that can be accessed for free at http://www.lirmed.com:5038/lm_dti.

## 1 Introduction

Identifying new drug-target interactions (DTIs) is a crucial step in a variety of biomedical and multi-pharmacological applications, such as drug discovery, drug relocation (Jarada et al., 2020), drug resistance, and side effect prediction (Masoudi-Nejad et al., 2013). Drug research and development is comprehensive, complex, and time-consuming, and the high experimental validation costs of the research and development processes of new drugs have plagued their success (Swinney and Anthony, 2011). Moreover, most small-molecule drugs have been approved by the FDA although they have multi-pharmacological properties and can interact with multiple target genes, which are not the main therapeutic target genes (i.e., the drugs have off-target effects) (Cichonska et al., 2015). A new trend caused by the off-target effect was to connect known drugs with the treatment of different diseases from those for which the drugs were originally developed for. With the continuous increase in official biomedical databases and the evolution of computational methods, it is more achievable to develop novel tools for predicting potential DTIs which would overcome the limitations of conventional experimental methods with respect to time and cost and help researchers find new, potentially beneficial, off-target effects of existing drugs. High probability DTIs of known drugs were predicted using computational methods which could be widely used in the field investigating their potential functions and underlying regulatory mechanisms, which can be a significant strategy for drug reusing (Chen et al., 2016).

According to recent reviews, several computational models have been proposed to predict DTIs. Early prediction methods for DTIs can be broadly classified into two categories: docking-based methods (Alonso et al., 2006; Cummings et al., 2007; Ma et al., 2013) and ligand-based methods (Lam et al., 2019). The docking methods consider the 3D structures of the target genes, which is extremely time-consuming, and the 3D structure information of all target proteins is difficult to obtain. In ligand-based methods, known ligands are compared with target proteins. However, the number of known ligands is reduced; thus, limiting the application of ligand-based methods.

The emergence of a large number of biological data sources, such as omics data, phenotypic groups, pharmaceutical chemical structures, and biological interactions, has promoted the development of various drug strategies for drug reuse, contributing to the development of new methods for discovering new DTIs according to the information of different target genes and drugs in the biological data sources. These methods utilise chemical and genomic information regarding target genes and drugs to construct computational models based on information networks, machine learning algorithms, and deep learning algorithms. Methods based on machine learning algorithms utilise feature engineering to represent drug-target pairs based on feature vectors (FV) which are extracted from structured data (Rayhan et al., 2017; Sachdev and Gupta, 2019). Methods based on deep learning algorithms construct a hierarchical representation of the data through multiple level layers of abstraction which has been proven effective in DTI prediction (Lee et al., 2019; Berrar and Dubitzky, 2021).

Conventional network-based methods for DTI prediction include the network-based inference (NBI) model (Cheng et al., 2012; Wu et al., 2016), path score model (PSM) (Olayan et al., 2018; Xuan et al., 2019; Thafar et al., 2020), and bipartite local model (BLM) (Bleakley and Yamanishi, 2009). For example, DDR (Olayan et al., 2018), a method based on the path score model, constructs a heterogeneous network composed of known drug target interactions, drug-drug similarities, and target-target similarities, whereas the path scores of different drug-target paths are fed into the random forest classifier to predict novel drug-target interactions. This method requires extensive network analysis and path scores between nodes, which are not always readily accessible or even unavailable. Therefore, more methods based on network embedding (Luo et al., 2017; Mohamed et al., 2020; Zeng et al., 2020; Alshahrani et al., 2021) have been proposed to overcome the limitations of conventional network analysis. Among these newly proposed methods, the nodes in the network embedding methods are represented by low-dimensional vectors which best preserve the structures and topology information of the networks. Neighbourhood regularised logistic matrix factorisation (NRLMF) (Liu et al., 2016) is a typical computational model based on matrix factorisation. NRLMF calculate the probability of each drug-target pair by applying logical matrix factorisation. Furthermore, logical matrix factorisation was combined with neighbourhood regularisation. The potential feature vectors representing the drugs and target genes were first extracted. Then, NRLMF uses the nearest neighbour of drug-drug similarity and target-target similarity to eliminate the noise of all similar neighbours. DTiGEMS+ (Thafar et al., 2020), which can be used to construct the same heterogeneous network as with DDR, generates the characteristics of drugs and targets using node embedding technology. Finally, these characteristics are input into a random forest classifier. DTi2Vec (Thafar et al., 2021) can be used to identify DTIs by using network representation learning algorithm and ensemble learning. It constructed a heterogeneous network and utilised the node2vec algorithm to gain the characteristics of each drug-target pair. TriModel (Mohamed et al., 2020) uses a knowledge graph (KG) to obtain KG embedding of the nodes and edges in a network by integrating multi-information sources. In this case, the DTI scores are calculated using the decomposition of the training tensor in the KG embedded in the TriModel. DNILMF (Hao et al., 2017) is a method based on similar network fusion (SNF) (Wang et al., 2014). It combines the similarity between drugs and targets with SNF, and DTIs are predicted according to the graph nearest neighbour of the drug-target pairs. Ro-DNILMF(Li et al., 2022) combines KG embeddings and DNILMF, and it achieves a better prediction performance than TriModel and DNILMF. MHSADTI (Cheng et al., 2022), an end-to-end deep learning method, predicts DTIs based on the graph attention network and multi-head self-attention mechanism. Supplementary Table S1 summarizes the aforementioned models in a tabular form.

However, conventional DTI prediction methods usually have a high false-positive rate which greatly limits their application. The main reason for this phenomenon is that the heterogeneous network adopted by these methods does not contain comprehensive information related to drugs and their targets, which makes graph-embedding methods unable to gain feature vectors with sufficient node information. Moreover, methods for extracting feature vectors should be further improved using advanced merging methods.

In this study, we proposed a novel model, LM-DTI, which constructs heterogeneous networks using related lncRNA and miRNA information and adopts a graph embedding algorithm, path scoring model, and ensemble learning technology to predict potential DTIs. The development of LM-DTI was primarily motivated by improving the prediction and avoiding the limitations of conventional methods. Four standard datasets and a large-scale DrugBank dataset were used, and the prediction performance of LM-DTI was verified using several network-based methods. The effectiveness of LM-DTI was confirmed using area under the precision-recall curve (AUPR) and area under the curve (AUC), and the novel DTIs were confirmed using reliable databases and scientific literature.

## 2 Materials and methods

### 2.1 Datasets

In this study, we utilised five datasets (Table 1) to evaluate the prediction performance of LM-DTI during the experimental phase. Four of these datasets were Yamanishi_08 (Yamanishi et al., 2008) which are generally recognised as ‘gold standard’ datasets containing three categories of information: known Human DTIs, drug-drug similarity, and target-target similarity. The DTIs data of these datasets were downloaded from KEGG BRITE (Kanehisa et al., 2006; Kanehisa et al., 2017), BRENDA (Schomburg et al., 2004), SUPERTARGET (Günther et al., 2008), and DrugBank (Wishart et al., 2008). The chemical structures of the KEGG ligands and drugs in the KEGG drug database were collected to calculate drug similarity (Hattori et al., 2010). The target amino acid sequences were obtained from the KEGG gene database, and the sequence similarities of the target genes used standardised Smith-Waterman (Smith et al., 2012) scores which calculated by the comparisons of related protein sequences. In Yamanishi_08 dataset, DTI information has often been categorised into the following four categories according to the target proteins, including nuclear receptor (NR) dataset, enzyme (E) dataset, ion channel (IC) dataset and G protein coupled receptor (GPCR) dataset.

**TABLE 1:** Benchmark Yamanishi_08 datasets and FDA_DrugaBank dataset statistics.

Statistics	Benchmark datasets				FDA_DrugBank
	NR	GPCR	IC	Enzyme	
Number of drugs	54	223	210	445	1,525
Number of targets	26	95	204	664	1,408
Known DTIs	90	635	1,476	2,926	9,874
Unknown DTIs	1,314	20,550	41,364	292,554	2,137,326

The FDA_DrugBank dataset includes the known DTIs data obtained from the DrugBank database, which consists of five types of data: the interaction data of lncRNA-target from lncRNA2Target (Cheng et al., 2019), miRNA-target data from miRTarBase (Huang et al., 2020), miRNA-drug association data from NRDTD (Chen et al., 2017) and SM2Mir3 (Liu et al., 2013), lncRNA-miRNA association data from NPInter v4.0 (Teng et al., 2020) and lncRNA-drug interaction data from D-lnc (Jiang et al., 2019), and NRDTD (Chen et al., 2017).

### 2.2 Similarity calculation

Drug-drug and target-target similarities were calculated and standardised into ranges (0, 1) in our study. For the four standard datasets in the Yamanishi_08 dataset, drug and target gene similarities were calculated based on the data from Nascimento et al. (2016). To calculate drug similarities, different chemical structure fingerprints, drug related gene ontology annotations, side effect spectra, and anatomical therapeutic chemical codes were used. Similar to drug-drug similarities, target-target similarities were calculated using different amino acid sequence maps of the targets, target protein function annotation of gene ontology terms, and protein-protein interaction networks.

For the FDA_DrugBank, we utilised the similarity data proposed by Olayan et al. (2018) who utilised the FDA_DrugBank to evaluate the effectiveness of DDR and calculated drug-drug similarities based on molecular fingerprints, drug interaction spectra, drug side effect spectra, drug spectra of anatomical therapy coding (ATC) system, drug-induced gene expression spectrum, and drug disease spectrum. The target-target similarities were calculated using the protein amino acid sequences, GO annotations, proximity of the PPI network.

### 2.3 Construction of the drug-target heterogeneous network

**FIGURE 1:**
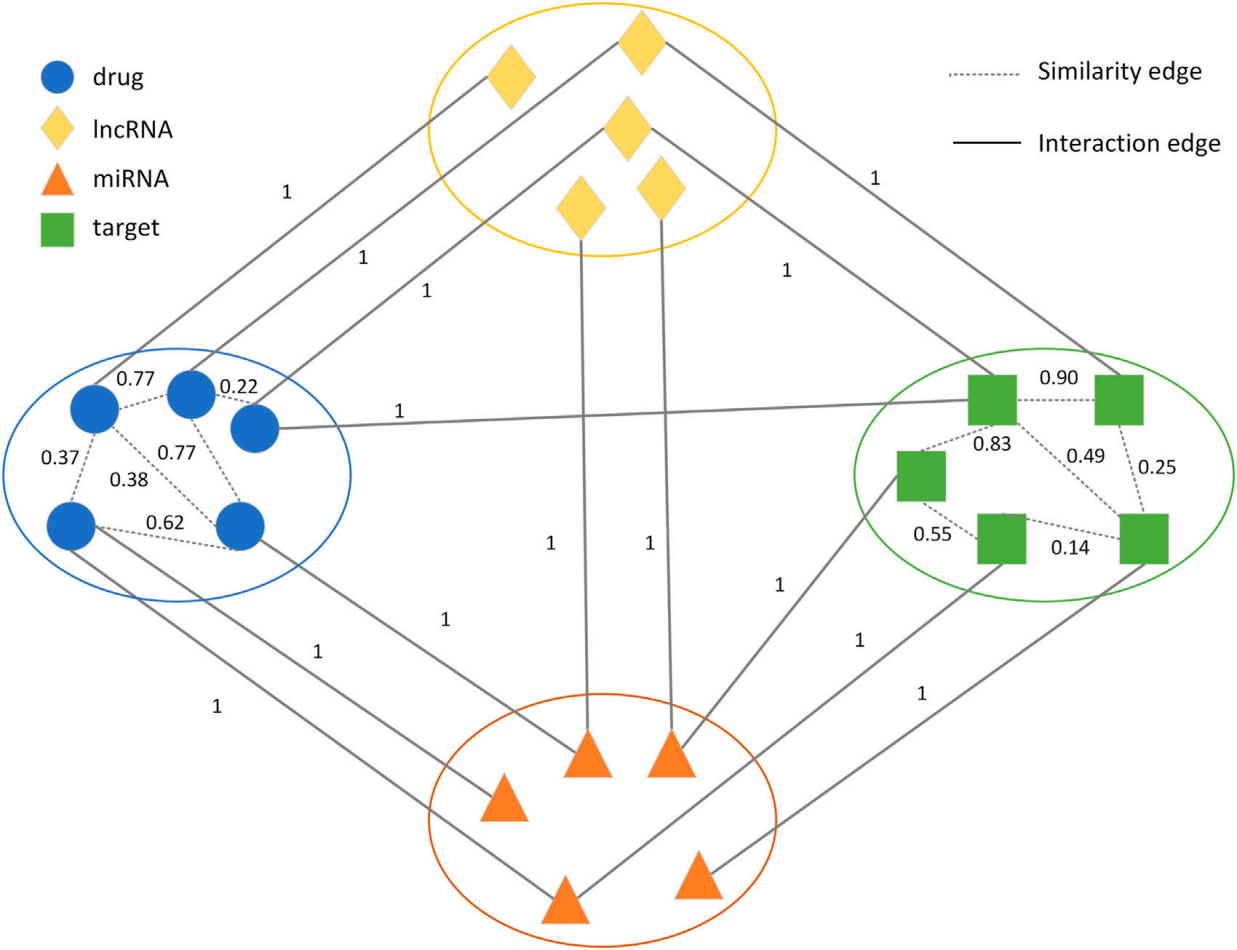
Schematic representation of the drug-target heterogeneous network.

A novel weighted heterogeneous network $G\left(V,E\right)$ with association networks between four biomolecules (drugs, target genes, lncRNAs, and miRNAs) was constructed and extended with drug-drug and target-target similarities from the FDA_DrugBank. $G\left(V,E\right)$ consisted of the lncRNA node list $L=\left\{l_{1},l_{2},...,l_{i}\right\}$ , drug node list $D=\left\{d_{1},d_{2},...,d_{n}\right\}$ , miRNA node list $M=\left\{m_{1},m_{2},...,m_{j}\right\}$ and target node list $T=\left\{t_{1},t_{2},...,t_{m}\right\}$ . There were two types of edges in $G\left(V,E\right)$ . One class represented the interactions between four types of nodes, which included drug-target, drug-lncRNA, drug-miRNA, lncRNA-target, miRNA-target, and lncRNA-miRNA interactions. The weights of the edges were 1. The other class represented drug-drug and target-target similarities, and the values of these similarities were introduced as edge weights which were between 0 and 1. Based on the weighted heterogeneous network, we solved the DTI prediction problem by predicting the unknown links in the heterogeneous network (Figure 1), which could improve the accuracy of the DTI prediction.

### 2.4 Construction of negative samples

All possible drug-target pairs were constructed, and we randomly selected a set of drug-target pairs as negative samples because there were not enough experiments to verify all drug-target pairs. According to the existing prediction methods, known DTIs are generally regarded as positive samples. The drug-target pairs corresponding to the positive samples were subsequently removed from the negative samples. Next, we employed different methods for extracting the characteristics of each drug-target pair. The feature vectors of the drug-target pairs are represented by $X=\left\{x_{1},x_{2},\ldots ,x_{n*m}\right\}$ and their labels are indicated by $Y=\left\{y_{1},y_{2},\ldots ,y_{n*m}\right\}$ , where n is the number of drugs and m is the number of targets. If the drug interacted with the target, the corresponding label value in Y was 1; otherwise, the label value was 0. In doing so, the issue addressed in our study was changed from predicting the potential drug-target associations to a binary classification problem based on the network path score, graph embedding algorithm, and ML methods.

### 2.5 Workflow of LM-DTI model

**FIGURE 2:**
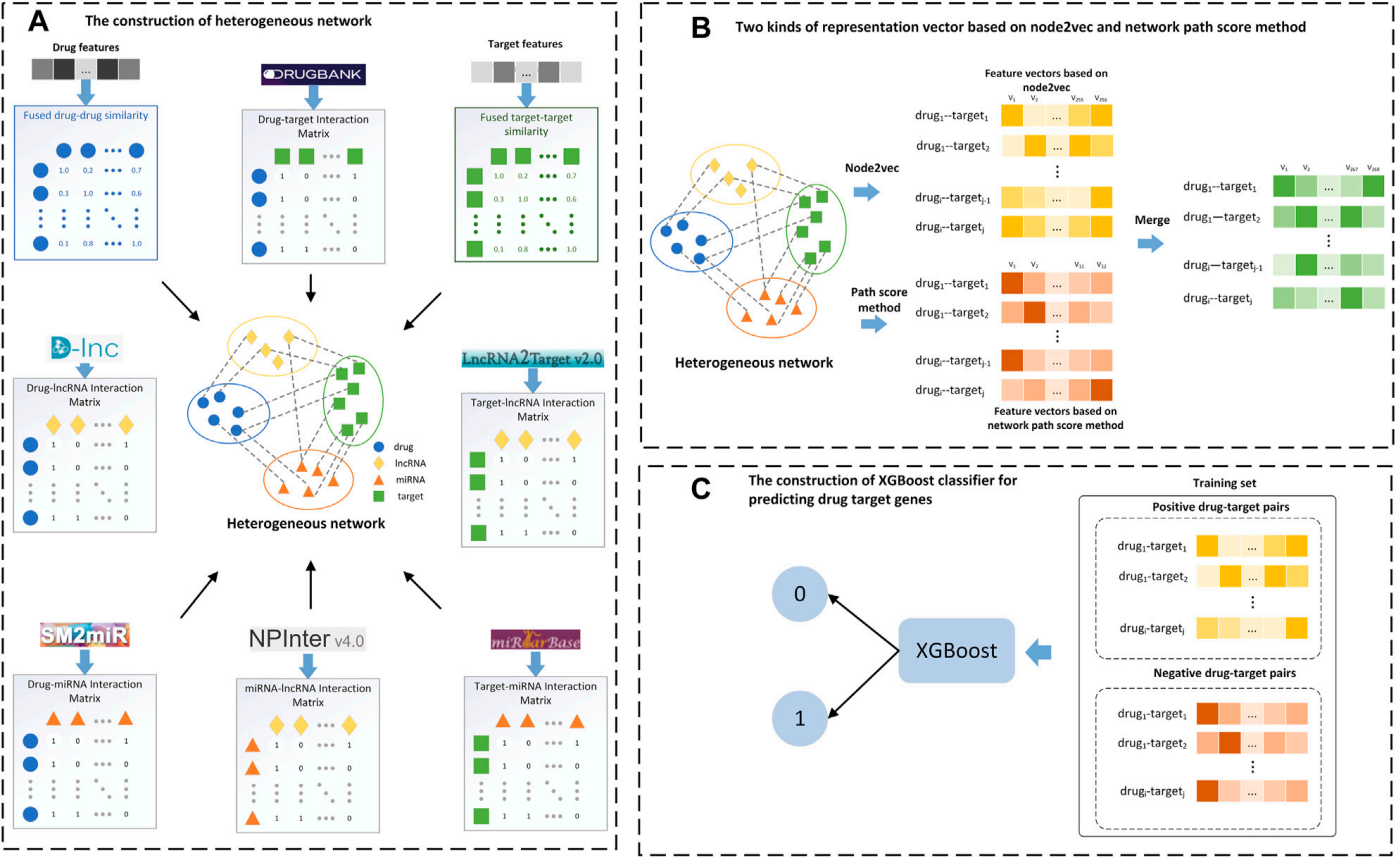
Flow chart of the LM-DTI model. **(A)** Filter the target-target and drug-drug similarity graphs to construct a full DTI network and generate the drug-target interaction matrix. **(B)** Apply the graph embedding algorithm on the full DTI network and calculate the network path score of each drug-target pair to generate the network path score vectors. **(C)** Input the feature vectors and the network path score vectors into the XGBoost classifier to obtain the class labels.

The main steps in implementing the LM-DTI are shown in Figure 2. First, the drug and target gene similarity data were pre-processed. Second, the heterogeneous network $G\left(V,E\right)$ was constructed using the drug-drug similarities, the target-target similarities and the interactions among drugs, target genes, lncRNAs and miRNAs. Third, the feature vectors were extracted using the graph embedding algorithm (node2vec) for drug and target nodes. Fourth, the network path score of each drug-target pair was calculated as feature vectors. Finally, the feature vectors and the calculated network path score vectors were fed into the ensemble learning classifier, XGBoost, and the prediction result was calculated as the class label of each drug-target pair.

### 2.6 Integration of similarities

The drug-drug or target-target similarity data were represented by similarity matrix $S=\left(s_{i,j}\right)$ , where $s_{i,j}$ is the similarity value between ${drug}_{i}$ and ${drug}_{j}$ or between ${target}_{i}$ and ${target}_{j}$ to indicate their similarity levels. The average (AVG), geometric average (GeoM), maximum (MAX), and latest similarity fusion algorithm SNF (Wang et al., 2014) were utilised to integrate the drug-drug and target-target similarity data. A similarity network was constructed using the SNF algorithm for each drug or each target, and the k-nearest neighbour (KNN) was used to integrate each similarity network with the information gained from other related networks. These networks were integrated iteratively. Finally, all similarity networks were integrated into a single network, at which time the SNF stopped operating.

### 2.7 Graph embedding methods for feature vector extraction

Several conventional protocols used the random walk method to improve the quality of feature extraction in heterogeneous networks (Su et al., 2018; Yue et al., 2020). The random walk method traverses a graph from one node or a series of nodes. At any node in a graph, the traverser walks to the neighbour node with probability 1-*a* and jumps to any node in the graph with probability *a* which is called the jump occurrence probability. After each walk, each node in the graph was accessed, and a probability distribution was obtained, which was used as the input for the next walk step, and the process was repeated. When certain pre-conditions were satisfied, the probability distribution tended to converge. Finally, a stable probability distribution was obtained.

In our model, node2vec (Grover and Leskovec, 2016), which further extended the DeepWalk (Perozzi et al., 2014) algorithm by changing the generation of random walk sequences, was utilised in network analysis and graph mining tasks. The DeepWalk algorithm selects the next node in a random walk sequence with uniform random distribution. Node2vec introduced width first search (BFS) and depth first search (DFS) into the generation process of a random walk sequence by adjusting two parameters, p and q . The parameter p controls the likelihood of a node being revisited immediately during the traversal. Instead, the parameter q controls the probability of revisiting a node or moving outward to a deeper node. BFS focused on adjacent nodes and characterised a relatively local network representation. The nodes in the BFS generally appear several times, thus reducing the variance of neighbouring nodes characterising the central node.

After constructing a weighted heterogeneous network graph $G\left(V,E\right)$ which contains four types of nodes (drugs, target genes, lncRNAs, and miRNAs), node2vec was used to transform all nodes from $G\left(V,E\right)$ into a vector space R_d_ with the following mapping function:

$$f:V\to R_{d},where\;d<\left|v\right|$$ (1)

Simultaneously, the structure and node similarities of the graph were retained based on the network topology. Multiple noce2vec hyperparameters were designed to improve the quality of feature extraction. The grid search method was introduced to adjust multiple parameters and obtain the best results in the cross-validation of each dataset. We tested the hyperparameter values as follows: return parameter, $p=\left\{0.25,0.5,1,2,4\right\}$ ; in-out parameter, $q=\left\{0.25,0.5,1,2,4\right\}$ ; the final output eigenvector dimension, $d=\left\{16,32,64,128\right\}$ ; and the number of random walks at each node, $num\_walk=\left\{5,10,15,20\right\}$ . The step length of the random walk is closely related to the size of the graph. In the FDA_DrugBank, the random walk steps from 60 to 120 were tested, increasing by 20 each time (i.e., 60, 80, 100, and 120). In the Yamanishi_08 NR, the random walk steps ranged from 10 to 40, increasing by 10 each time (i.e., 10, 20, 30, and 40). Supplementary Table S2 gives the optimised hyperparametric values for Yamanishi_08 and FDA_DrugBank dataset.

### 2.8 Path score method

Typically, the feature vectors of drug-target pairs are not sufficient to acquire the best prediction performance. LM-DTI uses network path scores to obtain extra feature vectors of drug-target pairs as supplemental feature information. Based on the heterogeneous graph $G\left(V,E\right)$ , some of the nodes included drug-drug similarities, target-target similarities, and drug-target interactions to reduce runtime and improve efficiency. The path score of each drug-target pair in the sub-graph was calculated as another form of the eigenvector (Ba-Alawi et al., 2016). The path scores were calculated as follows:

$$score\left(d_{i},t_{j}\right)=\sum_{p=1}^{n}{\prod \left(P_{weights}\right)}_{ij}$$ (2)

where $P=\left\{p_{1},p_{2},...,p_{n}\right\}$ is the path set of all drug-target pairs, $d_{i}$ denotes ${drug}_{i}$ and $t_{j}$ denotes ${target}_{j}$ . We restricted the path from one drug node to one gene node and path categories with lengths of 2 and 3 were utilised to reduce the cost of the calculation. Each drug node or target node would appear only once in a path. Therefore, we obtained six potential path structures: $P=\left\{P_{1},P_{2},P_{3},P_{4},P_{5},P_{6}\right\}$ . The two path types had a length of two, namely, $P_{1}:\left(D-D-T\right)$ and $P_{2}:\left(D-T-T\right)$ , and the remaining four path types had lengths of three, namely, $P_{3}:\left(D-D-D-T\right)$ , $P_{4}:\left(D-D-T-T\right)$ , $P_{5}:\left(D-T-D-T\right)$ , and $P_{6}:\left(D-T-T-T\right)$ . The meta path score, $score\left(d_{i},t_{j},h,q\right)$ was calculated as follows:

$$score\left(d_{i},t_{j},h,q\right)=\prod_{\forall e_{x}\in P_{q}}\left(w_{x}\right)$$ (3)

where h indicates the category of the path structure, q indicates the number of P,w indicates the edge weight of $P_{q}$ .

The sum of the meta-path scores of each path structure, $SumScore\left(d_{i},t_{j},h\right)$ , was calculated as follows:

$$SumScore\left(d_{i},t_{j},h\right)=\underset{\forall P_{q}\in R_{ijh}}{\sum }score\left(d_{i},t_{j},h,q\right)$$ (4)

where $R_{ijh}$ represents a set of paths between ${drug}_{i}$ and ${target}_{j}$ .

The maximum score of the meta-path of each path structure, $MaxScore\left(d_{i},t_{j},h\right)$ was calculated as follows:

$$MaxScore\left(d_{i},t_{j},h\right)={\mathrm{MAX}}_{\forall P_{q}\in R_{ijh}}\left(score\left(d_{i},t_{j},h,q\right)\right)$$ (5)

The $SumScore\left(d_{i},t_{j},h\right)$ and the $MaxScore\left(d_{i},t_{j},h\right)$ were calculated as the two characteristic vectors of each path structure. To reduce the runtime, we used 3D matrix multiplications to obtain the path scores. Therefore, drug-drug similarity, target-target similarity, and drug-target interaction data were converted into a graph adjacency matrix. The path scores were computed by the matrix multiplication method introduced in DASPfind. Conventional matrix multiplication can be performed for the total score features, where the resulting matrix represents includesthe total score features. For the maximum score features, 3D matrix multiplication obtained the score of each path, and the maximum value was selected instead of the summation. Supplementary Table S3 gives the matrix multiplications corresponding to each path structure and the semantics of all path structures.

### 2.9 Selection of classifier

Three popular classifiers with supervised ML models were introduced: the random forest (RF) classifier, Adaptive Boosting (AdaBoost) classifier, and extreme gradient boosting (XGBoost) classifier. The RF classifier is a bagging method which integrates multiple decision trees as a strong classifier. The Adaboost and XGBoost classifiers are generally used to enhance classifier performances. AdaBoost increased the flexibility of the classifier by using weighted majority voting and was implemented using scikit-learn. XGBoost utilises parallel tree boosting which improves the calculation speed. We also performed hyperparametric optimisation of the training data using 10-fold cross-validation for each classifier. XGBoost adjusted more hyperparameters than AdaBoost, including weighted regularisation parameters (such as lambda and alpha), tree construction algorithm, and sub-sample ratio. The graph embedding algorithm was used on the entire graph G, and the feature vectors were generated for each drug-target pair which was combined with the path score feature vector of each drug-target pair. The known DTIs were treated as positive samples whose labels were all set to 1, while the corresponding negative sample labels were set to −1. The feature vectors and labels of all drug-target pairs were input into these classifiers, and the outputs were regarded as the prediction results of the LM-DTI.

### 2.10 Evaluation metrics

In this study, the AUC and AUPR were used to evaluate the performance of the LM-DTI. For classifiers, precision refers to the ability to not mark negative samples as positive, and recall refers to the ability to identify all positive samples, as shown in Equations (6–8).

$$FPR=\frac{FP}{TN+FP}$$ (6)

$$Recall=TPR=\frac{TP}{TP+FN}$$ (7)

$$Precision=\frac{TP}{TP+FP}$$ (8)

Where TP denotes true positive, FP denotes false positive, TN denotes true negative, FN denotes false negative, FPR denotes the false positive rate and TPR (or Recall ) denotes true positive rate.

We constructed receiver operating characteristic (ROC) curves according to different TPR and FPR of various thresholds and then calculated the AUC values (i.e., the area under the ROC curve). Moreover, we constructed the PR curve based on different precision and recall values at different thresholds and calculated the AUPR values (i.e., area under the PR curve). For highly unbalanced data, AUC was generally considered to be an overly optimistic evaluation index, while AUPR could provide a better evaluation in this case of unbalanced data, which separated the prediction scores of real interactions from those of unknown interactions. Therefore, AUPR was adopted as the most important evaluation index. The error rate ( ER ) of the prediction structure and the relative error rate reduction ( ΔER ) were also introduced in our model, which are defined in Equations (9, 10)

ER=1−AUPR (9)

$$\Delta ER=\frac{\left(ER2-ER1\right)}{ER2}$$ (10)

A 10-fold cross-validation was adopted to evaluate the prediction performance of the LM-DTI. The AUC and AUPR values from the experimental tests were calculated, and the AUC as well as the AUPR average values were considered the final results.

## 3 Results

### 3.1 DTI prediction performance of LM-DTI

Unless otherwise specified, all experimental results presented in this section were obtained with a 10-fold cross validation. We divided the dataset into 11 parts, randomly selected 10/11 positive and negative samples to train and test the model with 10-fold cross validation, and used the remaining 1/11 samples as an independent validation dataset to prevent the model from overfitting. In 10-fold cross validation experiment, the training dataset was randomly divided into 10 subsets. One subset was selected from the 10 subsets as the test set in each fold, and the rest were used as the training set to train the model. Supplementary Table S4 gives the prediction results for the independent validation dataset. In our study, three graph embedding algorithms (LINE, node2vec, and Struc2vec) were introduced into LM-DTI and evaluated using the average AUPR and AUC values of 10-fold cross validations (unless otherwise specified). The AUPR and AUC values of the experimental results under the node representation vectors of different graph embedding algorithms and databases are shown in Table 2. The best evaluation results of the metrics on the different databases were in bold. Node2vec achieved the best performance in terms of DTIs prediction. Although LINE had the highest AUPR in the GPCR dataset, it did not perform well in the large-scale DrugBank dataset. For a comprehensive comparison, node2vec was chosen to generate representation vectors for drugs and targets.

**TABLE 2:** The AUC and AUPR values of prediction results with different graph embedding methods.

Model	Metric	Yamanishi_08 datasets				FDA_DrugBank
		NR	GPCR	IC	E	
LINE	AUC	0.89	**0.99**	**0.99**	0.98	0.91
	AUPR	0.87	**0.99**	**0.98**	0.96	0.87
Struc2vec	AUC	0.82	**0.99**	**0.99**	**0.99**	0.92
	AUPR	0.80	0.97	**0.98**	0.96	0.88
node2vec	AUC	**0.90**	**0.99**	**0.99**	**0.99**	**0.99**
	AUPR	**0.90**	0.98	**0.98**	**0.97**	**0.96**

### 3.2 Comparison among different classifiers

For each of the five datasets, RF, AdaBoost, and XGBoost classifiers were utilised to predict the DTIs, and the average AUPR and AUC values of 10-fold cross validations were used as the evaluation metric. The experimental results are displayed in Table 3. The XGBoost classifier achieved optimal prediction outcomes and its AUPR and AUC values are bolded in Table 3. Further, statistical and comparative analyses were performed. Compared with the XGBoost classifier, the AdaBoost classifier did not perform well when it contained unrelated features with a high level of noise. The XGBoost classifier was more robust because it had regularisation parameters and could reduce variance. In addition, one of the main advantages of the XGBoost classifier is its high computational efficiency associated with its parallel processing method. The XGBoost classifier was advantageous in the present study because of its rapid computational speed, low data requirements, and accurate training results; thus, making it superior to the RF and AdaBoost classifiers.

**TABLE 3:** The AUC and AUPR values of LM-DTI under different classifiers.

Classifier	Metric	Yamanishi_08 datasets				FDA_DrugBank
		NR	GPCR	IC	E	
RF	AUC	0.87	0.98	**0.99**	0.98	0.97
	AUPR	0.85	0.96	0.97	**0.97**	0.93
AdaBoost	AUC	0.87	0.98	**0.99**	0.96	0.90
	AUPR	0.87	0.97	0.97	0.93	0.86
XGBoost	AUC	**0.90**	**0.99**	**0.99**	**0.99**	**0.99**
	AUPR	**0.90**	**0.98**	**0.98**	**0.97**	**0.96**

### 3.3 Model contrast

After the model construction, we compared the proposed LM-DTI model with six state-of-the-art DTI models: DDR (Olayan et al., 2018), DNLMF (Hao et al., 2017), NRLMF (Liu et al., 2016), DTiGEMS+ (Thafar et al., 2020), TriModel (Mohamed et al., 2020), and DTi2Vec (Thafar et al., 2021) on the NR, GPCR, IC, E, and DrugBank datasets.

The AUPR and AUC values for the different models are shown in Table 4 and Table 5. LM-DTI achieved the highest AUPR and AUC values on five datasets and its performing was better than that of the other models. The best evaluation results of the metrics on the different databases were in bold. The AUPR values of LM-DTI for four datasets were 4% higher than those of the other models. In particular, LM-DTI significantly outperformed any of the six state-of-the-art DTI models on the large-scale DrugBank dataset.

**TABLE 4:** The AUC values of LM-DTI and the contrast models on different datasets.

Dataset				Names of contrast models			
	NRLMF	DNILMF	DDR	TriModel	DTiGEMS+	DTi2Vec	LM-DTI
NR	0.85	0.86	0.88	0.89	0.89	0.89	**0.90**
GPCR	0.93	0.93	0.93	0.88	**0.99**	**0.99**	**0.99**
IC	0.96	0.97	0.97	0.90	**0.99**	**0.99**	**0.99**
Enzyme	0.96	0.97	0.92	0.95	**0.99**	**0.99**	**0.99**
DrugBank	0.93	0.95	0.97	**0.99**	**0.99**	**0.99**	**0.99**

**TABLE 5:** The AUPR values of LM-DTI and the contrast models on different datasets.

Dataset				Names of contrast models			
	NRLMF	DNILMF	DDR	TriModel	DTiGEMS+	DTi2Vec	LM-DTI
NR	0.72	0.66	0.83	0.84	0.89	0.89	**0.90**
GPCR	0.71	0.70	0.79	0.80	0.86	0.90	**0.98**
IC	0.88	0.87	0.92	0.93	0.96	0.98	**0.98**
Enzyme	0.87	0.89	0.92	0.95	0.96	0.98	**0.98**
DrugBank	0.41	0.31	0.61	0.67	0.61	0.88	**0.96**

**FIGURE 3:**
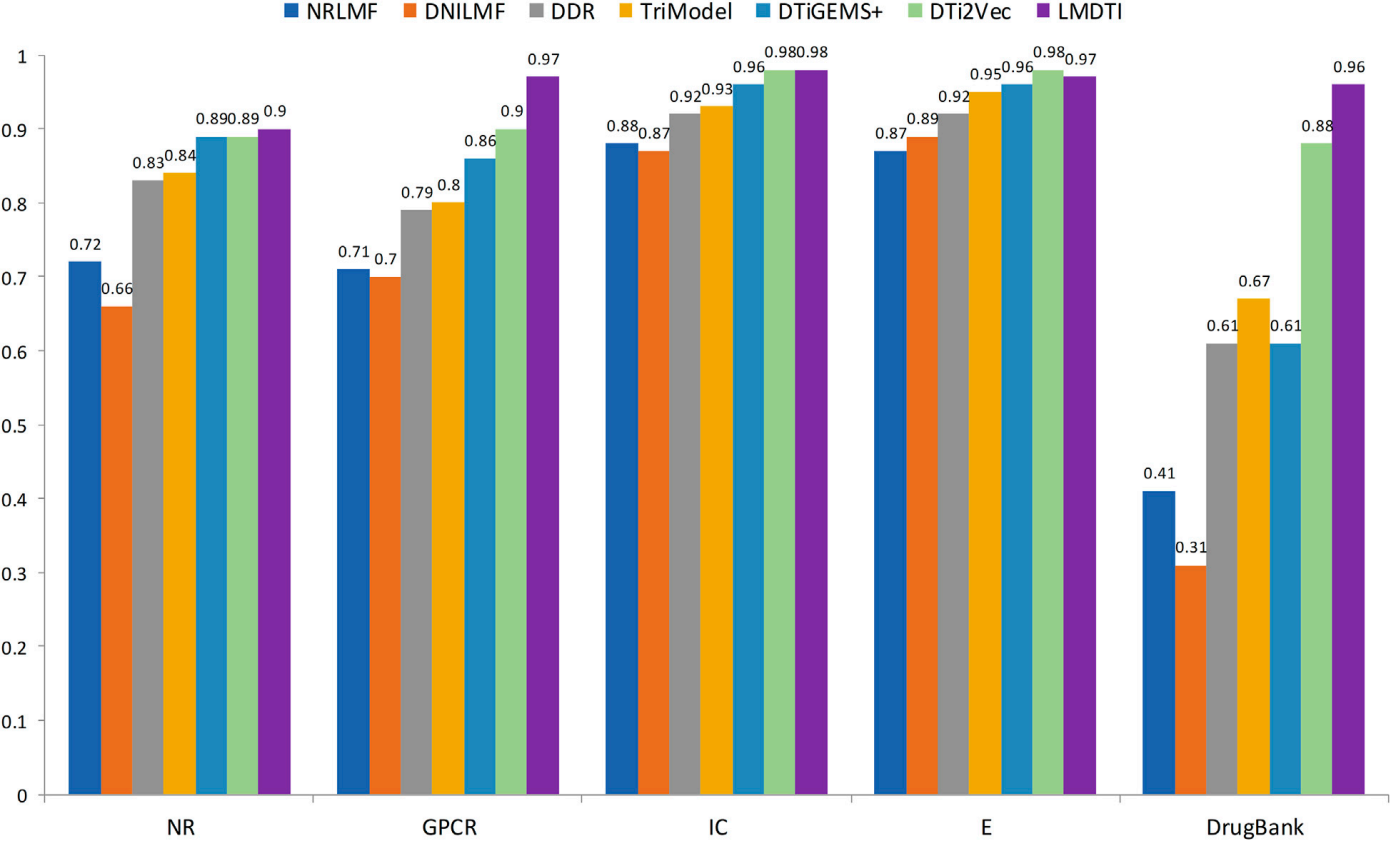
The AUPR values of LM-DTI and other advanced methods.

Figure 3 illustrates the prediction performance of the LM-DTI more intuitively. Supplementary Table S5 displays the results of each fold in the 10-fold cross-validation for each dataset. Compared to other models, the LM-DTI improved the accuracy of the DTI predictions. Moreover, the standard deviations of the LM-DTI on the four Yamanishi_08 and FDA_DrugBank datasets were 0.0447, 0.002, 0.0022, 0.0023, and 0.0012, respectively. These results indicate that LM-DTI exhibited high robustness. In addition, the evaluation metrics ( ER ; ΔER ) also reflected the improved performance associated with LM-DTI. Table 6 displays the ER and ΔER values of the LM-DTI and DTiGEMS + models on different datasets. LM-DTI was associated with a reduced relative error rate and also outperformed the DTiGEMS + model.

**TABLE 6:** The ER and ΔER values of LM-DTI and DTiGEMS + models on different datasets.

Dataset	ER values of LM-DTI (%)	ER values of DTiGEMS+ (%)	ΔER values (%)
NR	10.00	11.00	9.09
GPCR	2.00	14.00	85.71
IC	2.00	4.00	50.00
E	3.00	4.00	25.00
FDA_DrugBank	4.00	39.00	89.74

### 3.4 Ablation experiments

To verify the hypothesis that adding information related to lncRNAs and miRNAs could increase the feature information of network nodes and improve the accuracy of predicting drug-target associations, we obtained the feature vectors of drugs and target genes using the integrating network, and compared the prediction performances based on node2vec and PSM, respectively, or simultaneously. The AUC and AUPR values of the four groups of ablation experiments on the DrugBank dataset are listed in Table 7. The best evaluation results of the metrics of the four group ablation experimental results were in bold.

**TABLE 7:** The AUC and AUPR values of four group ablation experimental results.

Network setting	Feature extraction method	AUC	AUPR
integrating network	node2vec + PSM	**0.99**	**0.96**
integrating network	node2vec	0.98	0.94
original network	node2vec	**0.99**	0.82
original network	PSM	0.97	0.63

When node2vec and PSM were used simultaneously in the integrated network, the LM-DTI achieved a superior performance. For the original network, where lncRNA and miRNA nodes in heterogeneous networks were removed, the AUPR values of the predicted results were reduced, although the AUC values did not change significantly. If the node2vec or PSM method was used only for the integrating network or original network to extract feature vectors, the AUPR values were greatly reduced. Together, node2vec and PSM achieved optimal performance which indicates that the strategy of the model algorithm selection was reasonable.

### 3.5 Overview of LM-DTI server construction

A web server based on the LM-DTI model for lncRNA DTIs prediction was developed. The flask framework was used, with a back-end for data processing and calculation. At the front-end of the LM-DTI, the “HTML + CSS + Bootstrap” framework was used, whereas Ploty. js was used for graphical visualisation and JQuery was used for application logic. All computational algorithms were implemented in Python using the packages Numpy and Pandas. A total of 1,525 drugs and 1,408 targets were identified. LM-DTI is unrestricted (without a login procedure), compatible with most web browsers, and accessible at http://www.lirmed.com:5038/lm_dti.

**FIGURE 4:**
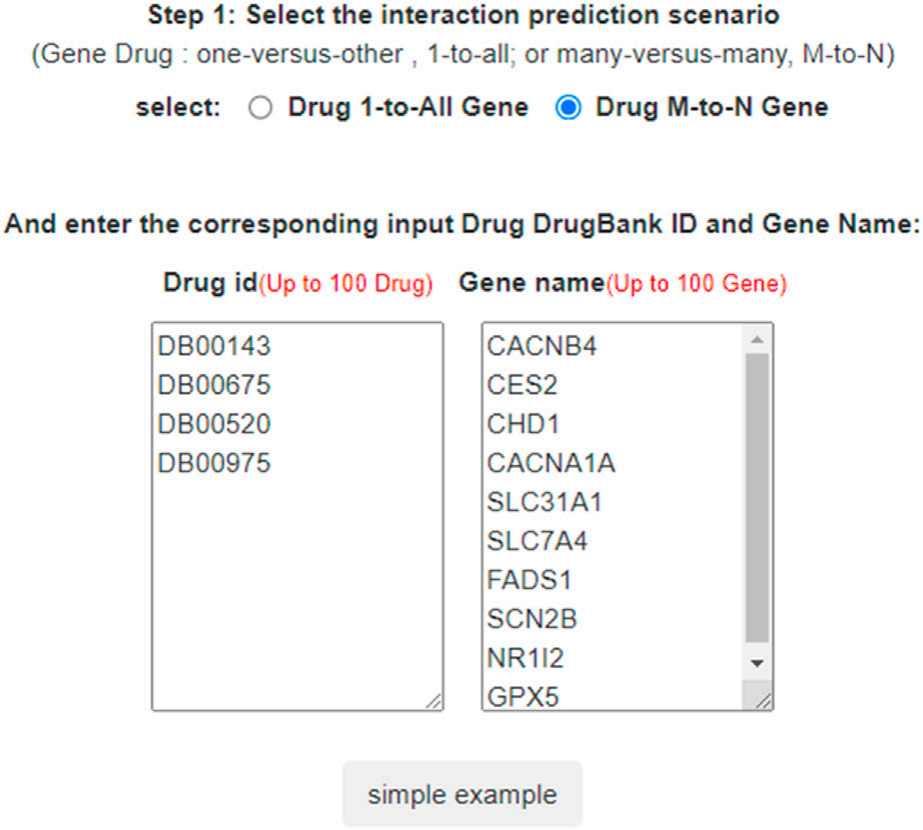
DTI predictions of LM-DTI.

**FIGURE 5:**
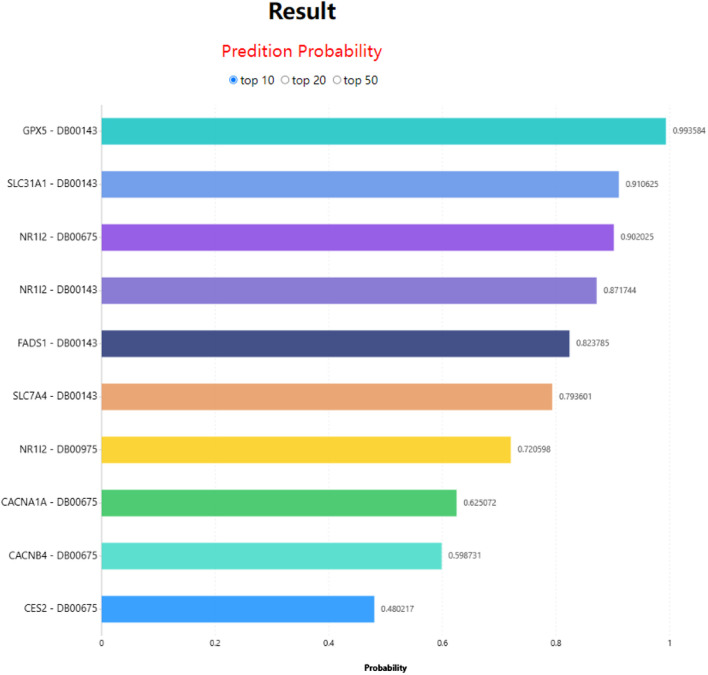
DTIs prediction results of LM-DTI.

In LM-DTI, users first submit a group of drugs and the targets of interest. Subsequently, users can choose to use LM-DTI to calculate the possibility of interaction between these drugs and targets. Users can only choose to submit one drug, and LM-DTI will calculate the possibility of interaction between the drug and all targets. As shown in Figure 4, the user first inputs a group of DrugBank IDs and gene names for the drugs and targets. If the drug names are not within the DrugBank IDs, the user must convert them to the DrugBank website or using other conversion tools. A simple example is provided for LM-DTI. As shown in Figure 5, LM-DTI can also visualise the results with one histogram chart, and users can select the top 10, 20, or 50 possible DTIs.

### 3.6 Case studies

To further demonstrate and analyse the effectiveness of LM-DTI, we selected drugs from five databases as case studies: Dyphylline from Enzyme, Clozapine from GPCR, Verapamil from IC, Tretinoin from NR, and Tamoxifen as well as Sorafenib from FDA_DrugBank. The DTIs related to these drugs were not included in the training set. Subsequently, LM-DTI calculated the possibilities of these drugs related to all targets and determined the prediction results. The top 10 targets for each drug are displayed in Table 8, and were most likely related to the drug according to the prediction scores. With the help of scientific literature and biomedical databases, such as KEGG and DrugBank, we manually confirmed these drug-target interactions. As shown in Table 8, there were a few predictive drug-target interactions which had not been established in the literature and biomedical databases. However, we believe that these interactions will be verified in the future.

**TABLE 8:** Case studies for LM-DTI.

Drug name	Target name	Validation evidence	Target name	Validation evidence
Dyphylline	PDE1C	KEGG: D00691	PDE4B	KEGG: D00691
Dyphylline	PDE8A	KEGG: D00691	PDE5A	unconfirmed
Dyphylline	PDE7A	KEGG: D00691	PDE8B	KEGG: D00691
Dyphylline	PDE7B	KEGG: D00691	PDE4A	KEGG: D00691
Dyphylline	PDE1A	KEGG: D00691	PDE11A	KEGG: D00691
Clozapine	DRD3	PMID: 31896438	CHRM1	PMID: 32593951
Clozapine	ADRA2A	KEGG: D00283	DRD2	PMID: 31496784
Clozapine	ADRA1B	PMID: 15695070	ADRA1D	unconfirmed·
Clozapine	HTR2A	KEGG: D00283	ADRA1A	unconfirmed·
Clozapine	ADRA2B	KEGG: D00283	HRH1	PMID: 27855565
Verapamil	CACNA1G	DrugBank: DB00661	CACNA1H	PMID: 18974361
Verapamil	KCNA7	PMID: 29743411	KCNA5	PMID: 30816676
Verapamil	CACNA1C	PMID: 20031608	KCNK5	PMID: 29743411
Verapamil	KCNA3	PMID: 19371328	KCNB2	PMID: 29743411
Verapamil	KCNA2	PMID: 7589202	KCNN4	PMID: 29743411
Tretinoin	RXRA	DrugBank: DB00755	RARB	KEGG: D00094
Tretinoin	RXRB	DrugBank: DB00755	RORB	unconfirmed
Tretinoin	RARA	KEGG: D00094	RORC	unconfirmed
Tretinoin	RARG	KEGG: D00094	NR0B1	unconfirmed
Tretinoin	RXRG	DrugBank: DB00755	RORA	unconfirmed
Tamoxifen	CYP3A4	PMID: 30909366	CYP2C19	PMID: 33432065
Tamoxifen	CYP2C9	PMID: 19935798	CYP2B6	PMID: 25940823
Tamoxifen	ABCB1	PMID: 29135105	CYP1A1	PMID: 23842721
Tamoxifen	ABCG2	PMID: 32087276	CYP1A2	PMID: 23412805
Tamoxifen	CYP3A5	PMID: 12419016	CYP2D6	PMID: 30909366
Sorafenib	CYP3A4	PMID: 30627802	CYP2C19	PMID: 21350850
Sorafenib	ABCG2	PMID: 28289864	CYP3A5	PMID: 21266595
Sorafenib	CYP2D6	PMID: 21350850	CYP2C8	PMID: 34765572
Sorafenib	ABCB1	PMID: 28289864	CYP2C9	PMID: 32700644
Sorafenib	CYP1A2	PMID: 33184472	CYP1A1	PMID: 24819355

The results of these case studies highlight the practical application value of LM-DTI, which could provide valuable candidates for subsequent experiments on drug-target interactions.

## 4 Conclusion and discussion

In this study we describe a novel prediction tool which solves the DTI prediction problem by predicting the unknown links based on heterogeneous networks. Our tool, LM-DTI, underutilises heterogeneous networks to predict potential DTIs instead of using isomorphic graphs. It integrates the drug similarity map, target gene similarity map, and the known interaction between drug, target gene, lncRNA, and miRNA to obtain a fully weighted heterogeneous network, $G\left(V,E\right)$ , the latter of which is an information-rich network that allows for improve predictive performance. We applied the node2vec algorithm to the heterogeneous network $G\left(V,E\right)$ for extracting an effective feature representation for each drug and target node, and the path score of each drug-target edge was subsequently calculated to expand the extracted features. We showed through a variety of experiments that this tool is highly efficient and practical and can be used to obtain the information necessary for DTI prediction. Compared with the six most advanced DTI prediction models by calculating multiple evaluation metrics, LM-DTI exhibited improved prediction performance. In addition, LM-DTI has proven its efficiency and reliability (based on AUPR) in predicting new DTI, which has been verified using multiple official databases and scientific literature.

One leading limitation of our tool is that the data of drugs and the corresponding targets are insufficient that restricted the number of target-drug interactions predicted by LM-DTI. It can be resolved in future work by expanding the number of drugs and targets contained in the LM-DTI. As future work, the prediction accuracy of LM-DTI should be improved by utilising different graph embedding algorithms and employing various types of drug-drug and target-target similarity data that may provide more useful information. One important extension of our research is that the heterogeneous network constructed in LM-DTI can also be extended to solve any biomedical problem based on heterogeneous networks, such as drug-miRNA association prediction, drug-lncRNA association prediction and protein-protein interaction prediction.

## Data Availability

The original contributions presented in the study are included in the article/Supplementary Material, further inquiries can be directed to the corresponding author.
