# Supplementary material for: LM-DTI: a tool of predicting drug-target interactions using the node2vec and network path score methods
Source: Front Genet. 2023 May 9;14:1181592. doi: 10.3389/fgene.2023.1181592 (PMC10203599; doi:10.3389/fgene.2023.1181592)
Supplement: Supplementary file 1 [file Table1.DOCX]

**Table S1** The Summarization of drug-target Prediction Models

| Categories | PMID | Model name | Introduction |
| --- | --- | --- | --- |
| Models based on path score model | 29186331 | DDR | A method based on the path score model, constructs a heterogeneous network composed of known drug target interactions, drug-drug similarities, and target-target similarities, whereas the path scores of different drug-target paths are fed into the random forest classifier to predict novel drug-target interactions. |
| Models based on matrix factorisation | 26872142 | NRLMF | NRLMF calculate the probability of each drug-target pair by applying logical matrix factorisation. |
| Models based on network embedding | 33431036 | DTiGEMS+ | DTiGEMS+ generates the characteristics of drugs and targets using node embedding technology, and these characteristics are input into a random forest classifier. |
|  | 34551818 | DTi2Vec | DTi2Vec can be used to identify DTIs by using network representation learning algorithm and ensemble learning. It constructed a heterogeneous network and utilised the node2vec algorithm to gain the characteristics of each drug-target pair. |
| Other prediction models | 31368482 | TriModel | TriModel uses a knowledge graph (KG) to obtain KG embedding of the nodes and edges in a network by integrating multi-information sources. In this case, the DTI scores are calculated using the decomposition of the training tensor in the KG embedded in the TriModel. |
|  | 28079135 | DNILMF | a method based on similar network fusion (SNF). It combines the similarity between drugs and targets with SNF, and DTIs are predicted according to the graph nearest neighbour of the drug-target pairs. |
|  | 36014371 | Ro-DNILMF | Ro-DNILMF combines knowledge graph embeddings and DNILMF. |
