# Supplementary material for: LM-DTI: a tool of predicting drug-target interactions using the node2vec and network path score methods
Source: Front Genet. 2023 May 9;14:1181592. doi: 10.3389/fgene.2023.1181592 (PMC10203599; doi:10.3389/fgene.2023.1181592)
Supplement: Supplementary file 2 [file Table2.DOCX]

**Table S2** Optimized hyperparametric values for each dataset

| Node2vec Parameters | NR | GPCR | IC | Enzyme | DrugBank |
| --- | --- | --- | --- | --- | --- |
| Number of features:  dimensions --d | 16 | 128 | 128 | 128 | 128 |
| Length of walk per source:  --walk length | 20 | 60 | 60 | 100 | 100 |
| Return hyperparameter:  -- p | 0.25 | 0.5 | 0.5 | 1 | 1 |
| In-out hyperparameter:  --q | 0.5 | 1 | 1 | 4 | 4 |
| Number of walks per source:  --num-walk | 10 | 10 | 10 | 10 | 10 |
