# Supplementary material for: LM-DTI: a tool of predicting drug-target interactions using the node2vec and network path score methods
Source: Front Genet. 2023 May 9;14:1181592. doi: 10.3389/fgene.2023.1181592 (PMC10203599; doi:10.3389/fgene.2023.1181592)
Supplement: Supplementary file 3 [file Table5.DOCX]

**Table S5** The results (AUPR value) of each fold in the ten-fold cross validation

|  | NR | GPCR | IC | Enzyme | DrugBank |
| --- | --- | --- | --- | --- | --- |
| Fold-1 | 0.978 | 0.977 | 0.974 | 0.971 | 0.956 |
| Fold-2 | 0.894 | 0.979 | 0.977 | 0.968 | 0.955 |
| Fold-3 | 0.963 | 0.979 | 0.975 | 0.974 | 0.958 |
| Fold-4 | 0.895 | 0.981 | 0.976 | 0.969 | 0.955 |
| Fold-5 | 0.858 | 0.981 | 0.976 | 0.971 | 0.959 |
| Fold-6 | 0.874 | 0.981 | 0.976 | 0.975 | 0.956 |
| Fold-7 | 0.829 | 0.979 | 0.974 | 0.974 | 0.956 |
| Fold-8 | 0.915 | 0.981 | 0.969 | 0.972 | 0.956 |
| Fold-9 | 0.851 | 0.979 | 0.975 | 0.970 | 0.957 |
| Fold-10 | 0.890 | 0.985 | 0.977 | 0.975 | 0.957 |
| AvgAUPR | 0.895 | 0.977 | 0.976 | 0.970 | 0.958 |
| std | 0.0447 | 0.0020 | 0.0022 | 0.0023 | 0.0012 |
