# Supplementary material for: LM-DTI: a tool of predicting drug-target interactions using the node2vec and network path score methods
Source: Front Genet. 2023 May 9;14:1181592. doi: 10.3389/fgene.2023.1181592 (PMC10203599; doi:10.3389/fgene.2023.1181592)
Supplement: Supplementary file 4 [file Table3.DOCX]

**Table S3** The matrix multiplication and the path structure

| **Path structure** | **Corresponding matrix multiplication** | **Length** |
| --- | --- | --- |
| D-->D-->T | DD_sim * DTI | 2 |
| D-->T-->T | DTI * TT_sim | 2 |
| D-->D-->D-->T | (DD_sim * DD_sim) * DTI | 3 |
| D-->T-->T-->T | DTI * (TT_sim * TT_sim) | 3 |
| D-->D-->T-->T | DD_sim * DTI * TT_sim | 3 |
| D-->T-->D-->T | DTI * DTI.Transpose * DTI | 3 |
