# Supplementary material for: LM-DTI: a tool of predicting drug-target interactions using the node2vec and network path score methods
Source: Front Genet. 2023 May 9;14:1181592. doi: 10.3389/fgene.2023.1181592 (PMC10203599; doi:10.3389/fgene.2023.1181592)
Supplement: Supplementary file 5 [file Table4.DOCX]

**Table S4** The prediction results for independent validation dataset

|  | NR | GPCR | IC | Enzyme | DrugBank |
| --- | --- | --- | --- | --- | --- |
| Accuarcy | 0.81 | 0.97 | 0.97 | 0.96 | 0.95 |
| Sensitivity | 0.79 | 0.96 | 0.96 | 0.96 | 0.96 |
| Specificity | 0.80 | 0.98 | 0.97 | 0.96 | 0.93 |
| F1 score | 0.79 | 0.97 | 0.97 | 0.96 | 0.95 |
| MCC | 0.61 | 0.93 | 0.93 | 0.92 | 0.90 |
| AUC | 0.91 | 0.99 | 0.99 | 0.99 | 0.98 |
| AUPR | 0.88 | 0.97 | 0.97 | 0.97 | 0.95 |
